# Supplementary material for: Plant-Specific Domains and Fragmented Sequences Imply Non-Canonical Functions in Plant Aminoacyl-tRNA Synthetases
Source: Genes (Basel). 2020 Sep 7;11(9):1056. doi: 10.3390/genes11091056 (PMC7564348; doi:10.3390/genes11091056)
Supplement: Supplementary file 1 [file genes-11-01056-s001.zip › revised supplementary files/Fig. S5.pdf]

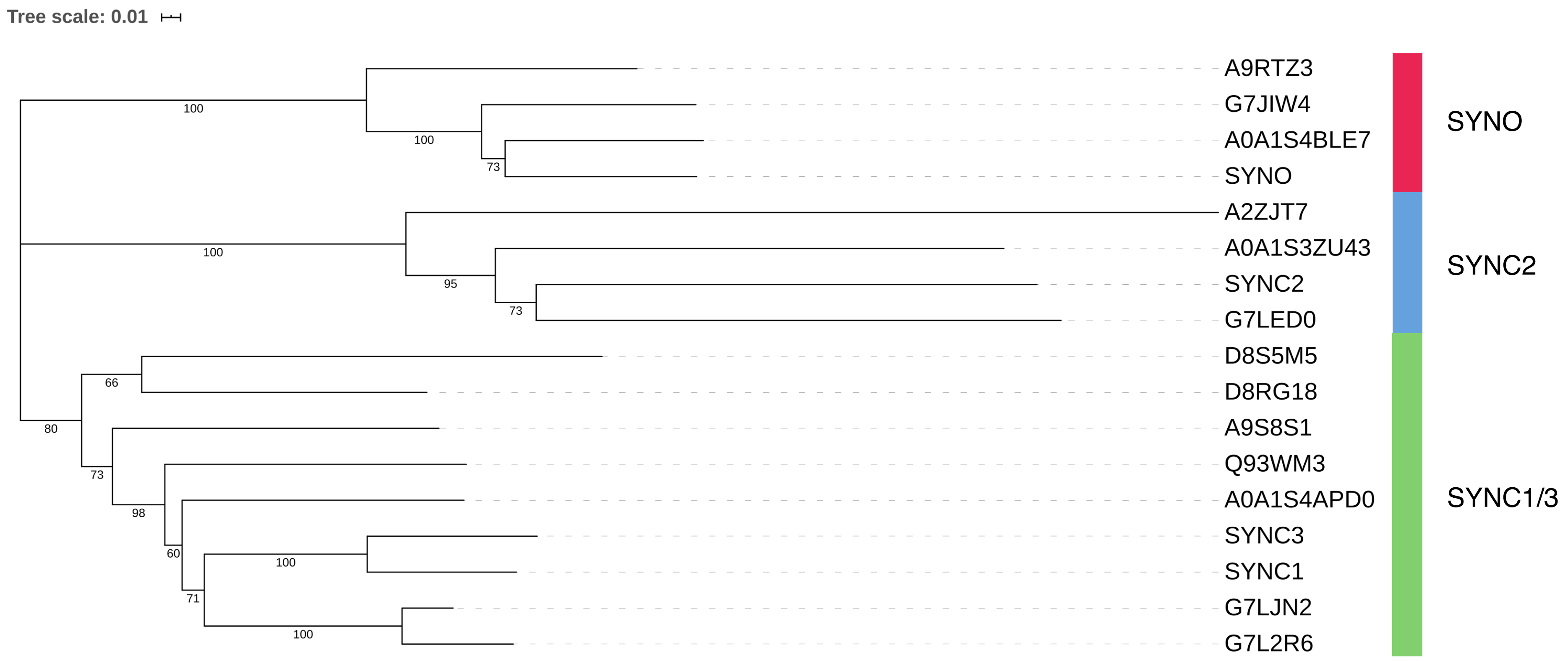

Fig. S5. Phylogenetic tree of plant AsnRS full-length sequences obtained from NJ method. AsnRS is classified as SYNC1/3 (cytosolic), SYNC2 (cytosolic), and SYNO (organellar). For sequences other than Arabidopsis AsnRSs, accession numbers assigned in UniProt database are shown. Bootstrap values from 1,000 replicates are shown in percentage.
