# Supplementary material for: Plant-Specific Domains and Fragmented Sequences Imply Non-Canonical Functions in Plant Aminoacyl-tRNA Synthetases
Source: Genes (Basel). 2020 Sep 7;11(9):1056. doi: 10.3390/genes11091056 (PMC7564348; doi:10.3390/genes11091056)
Supplement: Supplementary file 1 [file genes-11-01056-s001.zip › revised supplementary files/Table S2.pdf]

Table S2. Rice aaRSs identified in the database. Localization prediction and characteristic primary structures are noted. A total of seven fragmented sequences were identified all from class I.

ClassI	ID	length (aa)	localization prediction	primary structure	ClassII	ID	length (aa)	localization prediction	primary structure
ArgRS	Os01g0158500	598			AlaRS	Os01g0120400*	265	cytosol	
	Os05g0163000	606	cytosol			Os06g0245800	286	cp	
CysRS	Os03g0143300	137		C truncated‡		Os10g0182000	999	mt	
	Os09g0556500	569	mt, cp		AsnRS	Os01g0372700	559	cytosol	
	Os10g0463100	490	mt, cp			Os07g0485400	553	mt, cp	
GlnRS	Os01g0185200	796	cytosol			Os12g0414500	615	cytosol	
	Os05g0182800	496	cytosol		AspRS	Os01g0153400	384		
GluRS	Os01g0271200	715	cytosol			Os02g0139600	353	cytosol	
	Os02g0121000	567	cp			Os02g0686400	549	cytosol	
	Os10g0369000	713	cytosol		GlyRS	Os04g0398300	292		
IleRS	Os02g0183900	249	cytosol	C truncated‡		Os06g0103600	1068	cp	
	Os02g0778200	1058	cp			Os08g0538000	698	cytosol	
	Os06g0645400	849	cytosol		HisRS	Os02g0754700	487	mt, cp	
LeuRS	Os01g0120300	794				Os05g0150900	788	cytosol	
	Os05g0241100	322	cytosol	C truncated‡	LysRS	Os02g0623500	586	mt, cp	
	Os09g0378300	1094	cytosol			Os03g0586800	602	cytosol	
	Os09g0503400	1095	cytosol		PheRS	Os05g0558900	596	cytosol	
MetRS	Os01g0821800†	409	cytosol			Os10g0400800	493	cytosol	
	Os03g0209600	602	mt, cp			Os12g0533700	448	mt, cp	
	Os04g0303900†	259	cp		ProRS	Os07g0165000	377		
	Os06g0508700	89		N truncated§		Os12g0443700	510	cytosol	
	Os10g0400100	605	cytosol		SerRS	Os01g0559100	425	cytosol	
TrpRS	Os01g0743400	393	mt, cp			Os03g0198400	445	cytosol	
	Os07g0278400	107	cytosol	C truncated‡		Os11g0610900	509	mt, cp	
	Os12g0540900	408	cytosol		ThrRS	Os02g0538000	674	cp	
TyrRS	Os01g0500900	497	mt, cp			Os08g0295300	745	mt, cp	
	Os02g0722001	362	cytosol						
	Os04g0680550	332	cytosol						
	Os08g0150800	394	cytosol						
	Os08g0191800	357	cytosol						
	Os08g0320000	175	cytosol	C truncated‡					
ValRS	Os03g0111700	164	cytosol	C truncated‖					
	Os03g0335600	539	cytosol						
	Os03g0694900	1055	cytosol						
	Os07g0163500	958	cp						
	Os10g0506200	1050	cytosol						

*: AlaX ?
†: Arc ?
‡: Incomplete catalytic domain, KMSKS motif missing, Anticodon-binding domain missing
§: Catalytic domain missing
||: Catalytic domain missing, Anticodon-binding domain missing
