# Supplementary material for: Plant-Specific Domains and Fragmented Sequences Imply Non-Canonical Functions in Plant Aminoacyl-tRNA Synthetases
Source: Genes (Basel). 2020 Sep 7;11(9):1056. doi: 10.3390/genes11091056 (PMC7564348; doi:10.3390/genes11091056)
Supplement: Supplementary file 1 [file genes-11-01056-s001.zip › revised supplementary files/Table S3.pdf]

Table S3. Amino acid sequence identities (%) of WHEP domains among plant AsnRS and GlyRS. AsnRSs are denoted SYNC1, 2, and 3, while GlyRS as AtGRS.

	AT5G56680 (SYNC1)	*AT3G07420* (SYNC2)	*AT1G70980* (SYNC3)	*AT1G29880* (AtGRS)
AT5G56680 (SYNC1)		18.644	71.186	25.424
AT3G07420 (SYNC2)	18.644		18.644	16.949
AT1G70980 (SYNC3)	71.186	18.644		28.814
AT1G70980 (AtGRS)	25.424	16.949	28.814	
