# Supplementary material for: Plant-Specific Domains and Fragmented Sequences Imply Non-Canonical Functions in Plant Aminoacyl-tRNA Synthetases
Source: Genes (Basel). 2020 Sep 7;11(9):1056. doi: 10.3390/genes11091056 (PMC7564348; doi:10.3390/genes11091056)
Supplement: Supplementary file 1 [file genes-11-01056-s001.zip › revised supplementary files/Fig. S4.pdf]

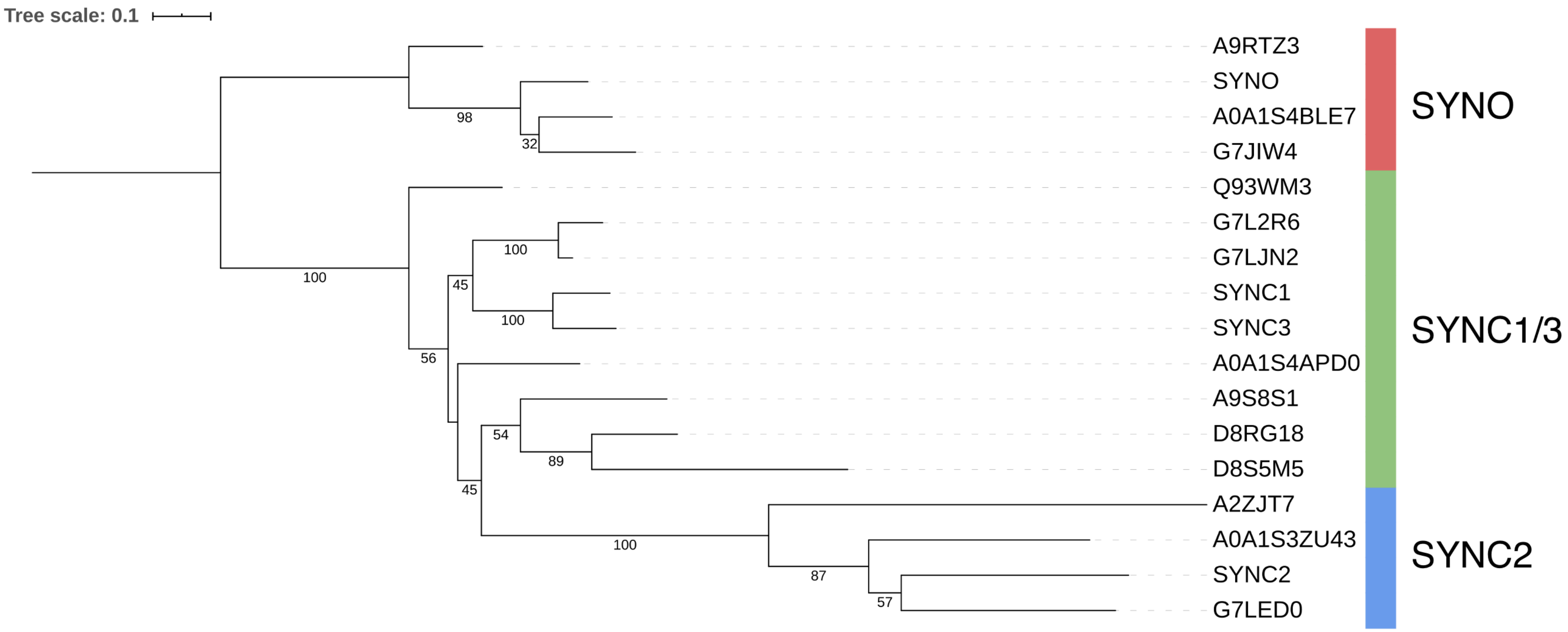

Fig. S4. Phylogenetic tree of plant AsnRS full-length sequences obtained using maximum likelihood method. AsnRS is classified as SYNC1/3 (cytosolic), SYNC2 (cytosolic), and SYNO (organellar). For sequences other than Arabidopsis AsnRSs, accession numbers assigned in UniProt database are shown. Bootstrap values from 1,000 replicates are shown in percentage. The major branches between SYNC1/3, SYNC2, and SYNO show significant bootstrap values indicating a clear distinction between these categories. Some branches within each category show relatively low values (< 75%).
