# Supplementary material for: Plant-Specific Domains and Fragmented Sequences Imply Non-Canonical Functions in Plant Aminoacyl-tRNA Synthetases
Source: Genes (Basel). 2020 Sep 7;11(9):1056. doi: 10.3390/genes11091056 (PMC7564348; doi:10.3390/genes11091056)
Supplement: Supplementary file 1 [file genes-11-01056-s001.zip › revised supplementary files/Fig. S6.pdf]

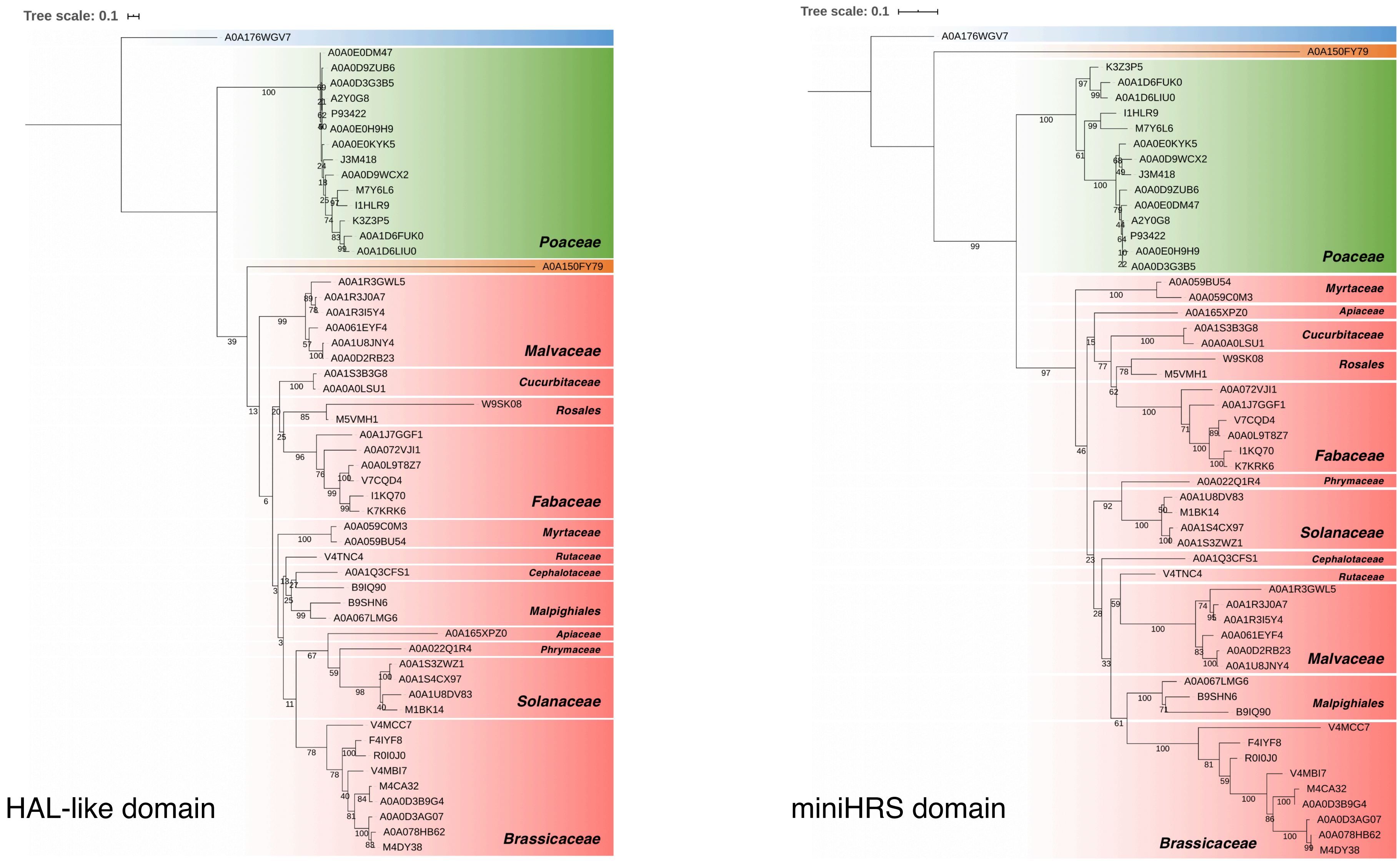

Fig. S6. Phylogenetic trees of HAL-like domain (left) and miniHRS domain (right) from plant HisRS obtained using maximum likelihood method. Branch colors represent taxonomic properties of the organisms: Dicotyledons (red), Monocotyledons (green), Bryophyte (blue), Pteridophyte (orange). Family names within each taxonomy are shown. Accession numbers assigned in UniProt database are indicated. Bootstrap values from 1,000 replicates are shown in percentage. The overall lower bootstrap values for HAL-like domain is perhaps attributed to a lower sequence identity of this domain. On the other hand, significant values are noted for miniHRS domain, especially for branches between different taxonomy. Nevertheless, both trees show similar pattern suggesting that both took the same evolutionary path.
