# Supplementary material for: Plant-Specific Domains and Fragmented Sequences Imply Non-Canonical Functions in Plant Aminoacyl-tRNA Synthetases
Source: Genes (Basel). 2020 Sep 7;11(9):1056. doi: 10.3390/genes11091056 (PMC7564348; doi:10.3390/genes11091056)
Supplement: Supplementary file 1 [file genes-11-01056-s001.zip › revised supplementary files/Table S1.pdf]

Table S1. The detailed information of maximum likelihood analysis for each phylogenetic tree.

Phylogenetic Tree	Number of taxa /number of sites for phylogenetic analysis	Selected amino acid substitution model	Log likelifood of ML analysis
AsnRS (Fig. 3c)	305/463	JTT+I+G4	-70776.76054
AsnRS (Supplementary Fig. S4)	17/550	LG+G4	-11159.22177
HisRS (Fig. 4c)	163/305	WAG+I+G4	-20335.48719
HAL-like domain (Supplementary Fig. S6)	54/378	JTT+I+G4+F	-18197.03712
miniHRS (Supplementary Fig. S6)	54/449	JTT+I+G4	-10484.50537
WHEP domain (Supplementary Fig. S3)	183/51	WAG+G4	-7999.436317
WHEP domain (Supplementary Fig. S1)	16/61	LG+G4	-1559.710407
