# Supplementary material for: Plant-Specific Domains and Fragmented Sequences Imply Non-Canonical Functions in Plant Aminoacyl-tRNA Synthetases
Source: Genes (Basel). 2020 Sep 7;11(9):1056. doi: 10.3390/genes11091056 (PMC7564348; doi:10.3390/genes11091056)
Supplement: Supplementary file 1 [file genes-11-01056-s001.zip › revised supplementary files/Fig. S3.pdf]

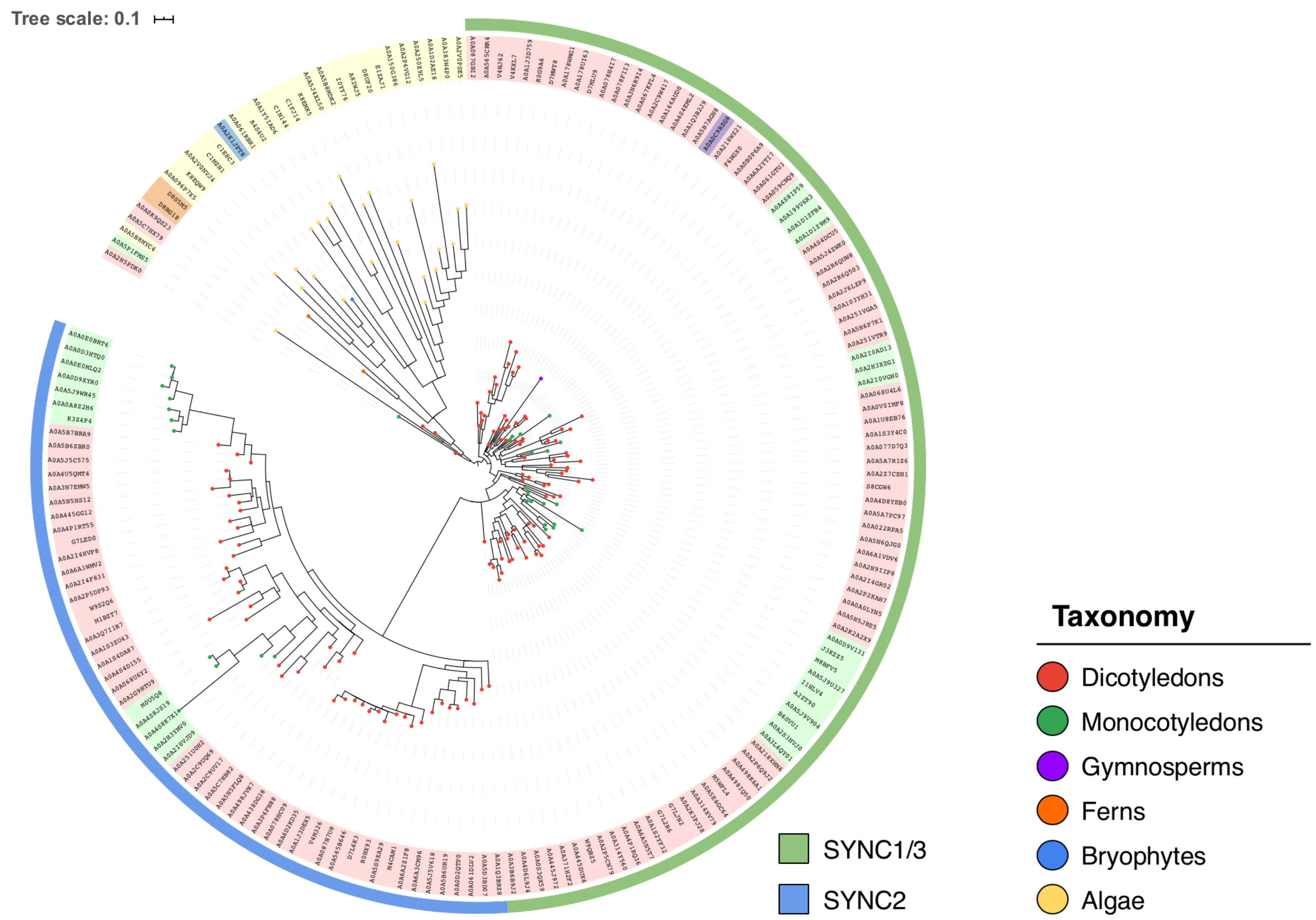

Fig. S3. Phylogenetic tree of WHEP domain sequences from plant AsnRS. SYNC1/3 are indicated with green boxes, while SYNC2 are indicated with blue boxes. Sequences from different taxonomy are colored differently. Accession numbers assigned in UniProt database are indicated. The WHEP domain phylogeny follows that of the full-length AsnRS phylogeny, and a clear distinction is seen in WHEP domain sequence of SYNC2 from that of SYNC1/3.
