# Supplementary material for: Plant-Specific Domains and Fragmented Sequences Imply Non-Canonical Functions in Plant Aminoacyl-tRNA Synthetases
Source: Genes (Basel). 2020 Sep 7;11(9):1056. doi: 10.3390/genes11091056 (PMC7564348; doi:10.3390/genes11091056)
Supplement: Supplementary file 1 [file genes-11-01056-s001.zip › revised supplementary files/Fig. S2.pdf]

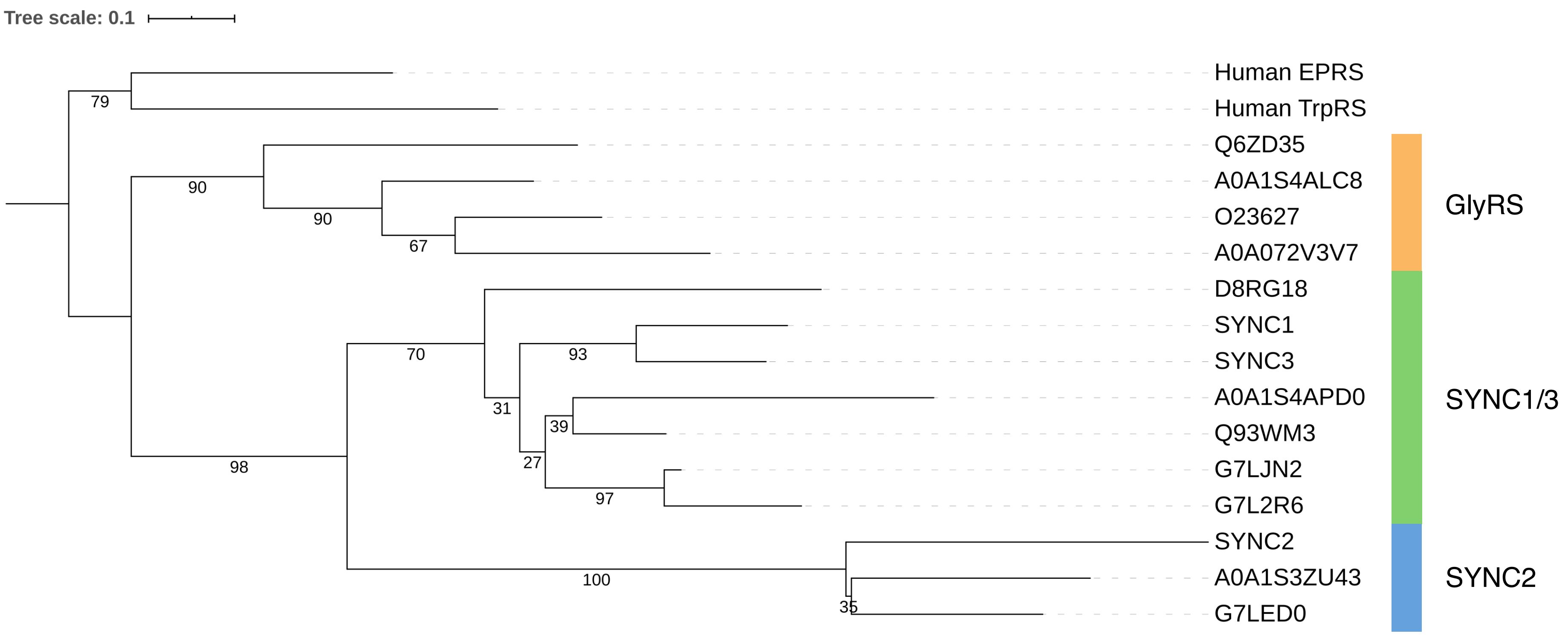

Fig. S2. Phylogenetic tree of WHEP domain sequences from human aaRSs and plant AsnRS and GlyRS obtained from NJ method. Plant AsnRSs are denoted SYNC1, 2, and 3. WHEP domain from human TrpRS and GluProRS (EPRS) are included. Others indicate accession numbers assigned in UniProt database. Bootstrap values from 1,000 replicates are shown in percentage.
