# Supplementary figures and images for: Plant-Specific Domains and Fragmented Sequences Imply Non-Canonical Functions in Plant Aminoacyl-tRNA Synthetases
Source: Genes (Basel). 2020 Sep 7;11(9):1056. doi: 10.3390/genes11091056 (PMC7564348; doi:10.3390/genes11091056)

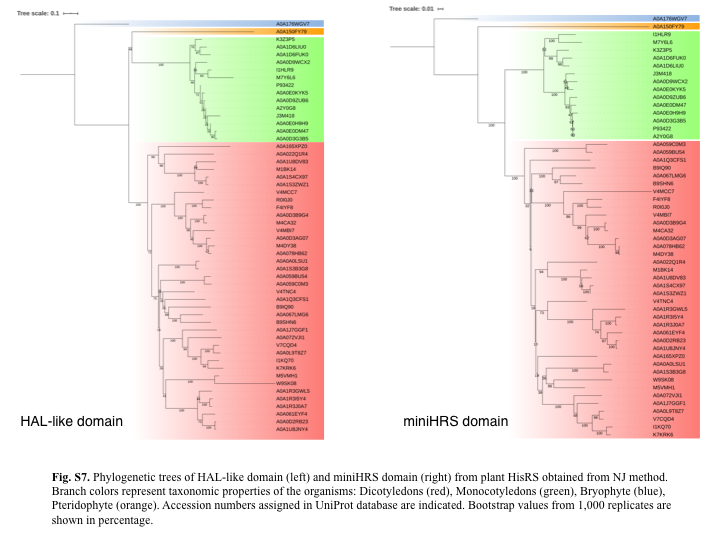

Supplement: Supplementary file 1 [file genes-11-01056-s001.zip › revised supplementary files/Fig. S7.png]
